# Gold nanoclusters conjugated berberine reduce inflammation and alleviate neuronal apoptosis by mediating M2 polarization for spinal cord injury repair

**DOI:** 10.1093/rb/rbab072

**Published:** 2021-12-02

**Authors:** Zipeng Zhou, Dan Li, Xiangyi Fan, Yajiang Yuan, Hongyu Wang, Dahao Wang, Xifan Mei

**Affiliations:** 1 Department of The First Clinical College, Liaoning University of Traditional Chinese Medicine, No. 79, Chongshan East Road, Huanggu District, Shenyang City, Liaoning Province 110847, P.R. China; 2 Department of Orthopedics, First Affiliated Hospital of Jinzhou Medical University, Jinzhou, China; 3 Department of Basic Science, Jinzhou Medical University, Jinzhou, China; 4 Department of Otolaryngology-Head and Neck Surgery, First Affiliated Hospital of Jinzhou Medical University, Jinzhou, China; 5 Jinzhou Medical University, No.40, Section 3, Songpo Road, Linghe District Jinzhou City, Liaoning Province 121001, P.R.China

**Keywords:** gold nanoclusters, berberine, macrophages/microglia, spinal cord injury

## Abstract

Spinal cord injury (SCI) leads to nerve cell apoptosis and loss of motor function. Herein, excessive activation of the M1 phenotype macrophages/microglia is found to be the main reason for the poor prognosis of SCI, but the selective activation phenotype (M2) macrophages/microglia facilitates the recovery of SCI. Thereafter, we used gold nanoclusters loaded berberine (BRB-AuNCs) to reduce inflammation by inhibiting the activation of M1 phenotype macrophages/microglia, which simultaneously inhibited neuronal apoptosis after SCI. *In vitro* and *in vivo* experiments showed that BRB-AuNCs reduced M1 protein marker CD86, increased M2 protein marker CD206, reduced inflammation and apoptotic cytokines (IL-1β, IL-6, TNF-α, Cleaved Caspase-3 and Bax). These results indicate that BRB-AuNCs have excellent anti-inflammatory and anti-apoptotic effects by inducing the polarization of macrophages/microglia from M1 phenotype to M2 phenotype. Thereafter, the motor functions of SCI rats were significantly improved after treatment with BRB-AuNCs. This work not only provides a new way for the treatment of SCI but also broadens BRB utilization strategies.

## Introduction

Spinal cord injury (SCI) has a devastating effect on people’s life quality [1–3]. The inflammatory cytokines induced by SCI evolved into secondary injury [4], which have caused serious complications of spinal diseases [5]. Meanwhile, nerve cell apoptosis induced by inflammatory response caused motor dysfunction. Effectively inhibiting the production of inflammatory cytokines can reduce nerve cell apoptosis and improve the prognosis of SCI [6].

As the main immune cells in the spinal cord, macrophages/microglia respond quickly after SCI [7]. Studies have found that macrophages/microglia are rapidly activated and polarized after SCI [8]. Activated microglia will polarize into two subtypes, M1 and M2 [9]. M1 type activated microglia after SCI exhibited obvious pro-inflammatory and neurotoxic effects, which aggravated the damage process, while M2 type cells exhibited anti-inflammatory and neuroprotective effects and promoted the recovery of injured tissues [10]. Therefore, finding a way to promote the polarization of M1–M2 will help the prognosis of SCI.

Many drugs have been explored to treat SCI, but the recovery efficacy is insufficient [11]. And low recovery efficiency may bring serious inflammations and infections, which may cause serious side effects [12, 13]. Berberine (BRB) is a quaternary ammonium alkaloid isolated from Coptis Rhizoma [14, 15], which is a positively charged herbal medicine [16]. As a natural product, BRB is known for its low side effects for reducing inflammations. However, the poor absorption rate limits their potential for rapid therapy of diseases [17]. Effective improvement of the bioavailability of BRB may evacuate their potential to inhibit various inflammations, though an effective enough strategy has been found.

In recent years, gold nanoclusters (AuNCs) have attracted wide attention from researchers. AuNCs are ultra-small nanomaterial (<3 nm) with excellent biocompatibility and have been used as a carrier to improve the biocompatibility of anticancer drugs [18–20], but they have never been used to magnify the efficiency of BRB for recovery of SCI.

**Figure 1.**
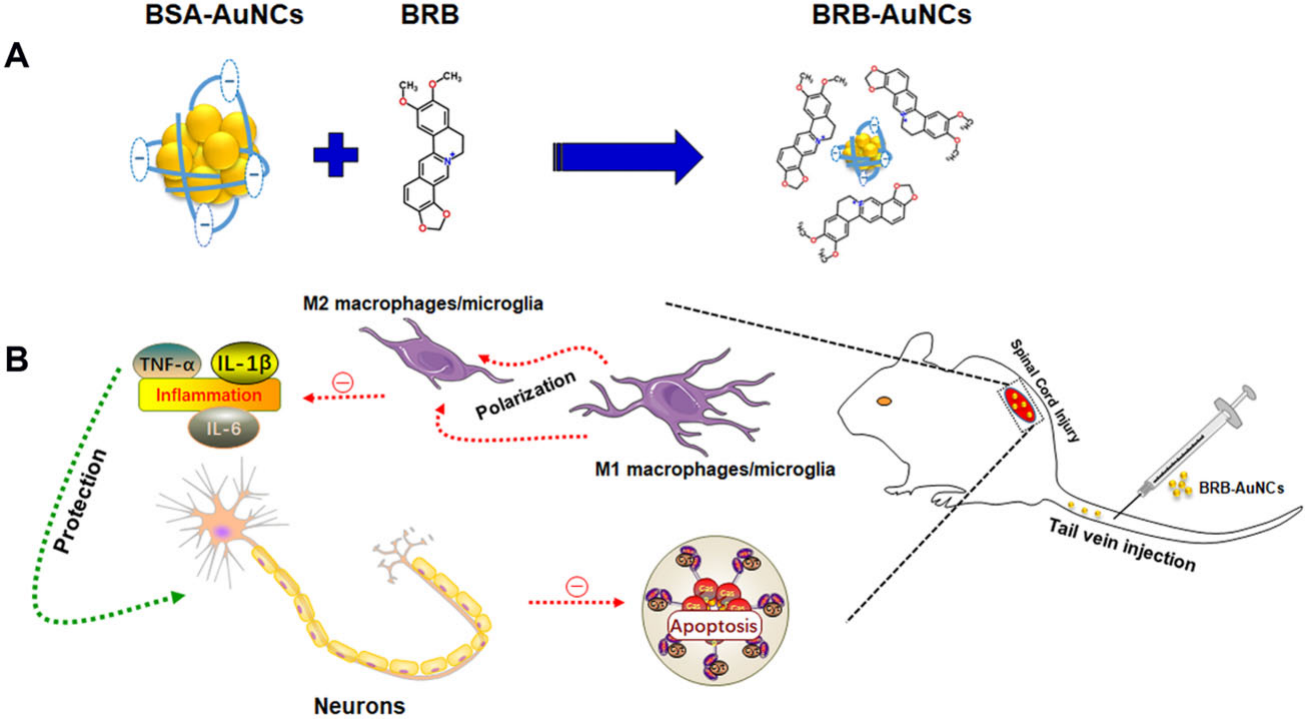
(**A**) Mechanism for the facile fabrication of conjugated BRB-AuNCs by the electrostatic interaction between BRB and negative charged BSA-AuNCs; (**B**) injection of BRB-AuNCs and modulation of M1/M2 macrophage and reduction of apoptosis after SCI

In this study, BRB was facilely conjugated to AuNCs as a new drug for SCI treatment (Fig.  1). AuNCs stabilized by negatively charged bovine serum albumin (BSA) are conjugating to positively charged BRB through electrostatic interaction. The conjugated drug, i.e. gold nanoclusters loaded berberine (BRB-AuNCs), showed remarkable effects for reducing inflammation, relieving neuronal apoptosis and promoting neuronal function recovery *in vivo* and *in vitro*. BRB-AuNCs were also found to recover the neurological function after SCI, with a significantly enhanced efficiency than BRB alone. BRB-AuNCs were found to reduce inflammation during secondary injury by promoting the polarization of the M1 phenotype to the M2 phenotype. At the same time, nerve cell apoptosis was inhibited and the recovery of motor function was promoted. Then, the SCI rat was fast cured with excitingly high efficiency. This work opens a new avenue for the fast treatment of SCI by BRB-AuNCs. It also provides a facile strategy by using AuNCs to improve the efficiency of BRB, so that natural products from herbs are promising to cure diseases quickly.

## Materials and methods

### Materials

Dulbecco’s modified Eagle’s medium (DMEM) and fetal bovine serum (FBS) were purchased from Gibco (USA). BRB, 3-(4,5-dimethylthiazol-2-yl)-2,5-diphenyl tetrazolium bromide (MTT), dimethyl sulfoxide (DMSO), HAuCl_4_. 3H_2_O (≥99.9% trace metals basis), BSA, Triton X-100 and lipopolysaccharide (LPS) were purchased from Sigma-Aldrich (USA). RIPA lysis buffer, phenylmethanesulfonyl fluoride, hematoxylin-eosin (HE) staining kit and Nissl staining solution (methylene blue) were obtained from Solarbio (China). The antibodies to anti-CD86, anti-CD206, anti-Cleaved Caspase-3 and anti-TNF-α were purchased from Cell Signaling Technology (USA). Anti-Bax, anti-Bcl-2, anti-IL-1β and anti-IL-6 were purchased from Affinity (USA). Anti-β-Tubulin, anti-GAPDH, HRP Affinipure Goat Anti-Mouse IgG and HRP Affinipure Goat Anti-Rabbit IgG were purchased from Proteintech (USA). The Alexa Fluor^®^568 goat anti-mouse/rabbit IgG and Alexa Fluor^®^488 goat anti-mouse/rabbit IgG were purchased from Invitrogen (USA). 4,6-dimethyl-2-phenylindole (DAPI) was purchased from Abcam (UK). RAW 264.7 cells and VSC 4.1 cells were obtained from the American type culture collection.

### Preparate of AuNCs and BRB-AuNCs

AuNCs stabilized by BSA were synthesized based on previous reports with slight modifications [21]. HAuCl_4_ solution (5 ml) (10 mM) was combined with 5 ml of BSA solution (50 mg/ml). After stirring evenly, 0.5 ml of NaOH solution (1 M) was injected with vigorously stirring. After that, the mixture was transferred to a water bath and kept at 37°C for 12 h. The product was dialyzed using a dialysis tube of 10 000 molecular weight cut-off against deionized water. Next, the product was filtered with a 0.22 μm syringe filter. One milligram of BRB was mixed with 1 ml of BSA-AuNCs and the mixture was kept overnight until a transparent solution was obtained. The large precipitates were separated by centrifugation at 3000 rpm. The drug encapsulation efficiency (EE) and load capacity (LC) were calculated using the following formula.

(1) $$\mathrm{EE}\%=\frac{\mathrm{weight}\;\mathrm{of}\;\mathrm{drug}\;\mathrm{in}\;\mathrm{NPs}}{\mathrm{weight}\;\mathrm{of}\;\mathrm{total}\;\mathrm{drug}}\times 100\%$$

(2) $$\mathrm{LC}\%=\frac{\mathrm{weight}\;\mathrm{of}\;\mathrm{drug}\;\mathrm{in}\;\mathrm{NPs}}{\mathrm{weight}\;\mathrm{of}\;\mathrm{NPs}}\times 100\%.$$

### Characterization of the materials

The morphology of AuNCs was observed by transmission electron microscope (TEM, Tecnai G2 F30 S-Twin, USA). The size and zeta potential of the materials were analyzed by Nano Measure and laser particle size analyzer (Zetasizer NANO ZS90, USA). The optical characteristic of the materials was tested by a UV–Vis spectrophotometer (UV-360, Japan) and a fluorospectrophotometer (RF-6000, China).

### Drug release *in vitro*

Five milliliters of BRB-AuNCs was placed in a dialysis bag (molecular weight cut-off, 8 kDa). The sealed dialysis bag was infused into a 30 ml release medium (PBS, pH = 7.4) and incubated with gentle shaking (100 rpm) at 37°C. At predetermined time points, 1 ml of culture medium was collected and stored at −20°C for further analysis. The remaining medium was removed and replaced with pre-warmed fresh PBS. The collected samples were quantified by High Performance Liquid Chromatography (HPLC, Shimadzu LC-2030, Japan).

### Biodistribution studies

Sprague Dawley rats were divided into two groups and received 10 mg/kg of BRB and BRB-AuNCs via tail vein injection after the SCI model had been established. The rats were euthanized by overdose anesthesia after 24 h. The spinal cord, heart, liver, spleen, lungs and kidneys were collected as soon as possible on the ice. All tissues were weighed and quickly frozen in liquid nitrogen and stored at −80°C until further processing. HPLC was used to determine the concentration of BRB in each sample.

### Cell culture

RAW 264.7 cells (mouse mononuclear macrophage leukemia cells) and VSC 4.1 cells (spinal anterior horn motor neuron tumor cell line) were cultured in DMEM-medium containing 10% FBS. The culture environment was kept in humid air containing 5% CO_2_ at 37°C. The media was changed every 3 days. LPS (100 ng/ml) was used to stimulate the inflammations *in vitro*. After the treatment with LPS for 24 h, the fresh media will be replaced in all groups. The cells were randomly divided into five groups: (i) control group: no further treatment was added to the cell culture mediums; (ii) LPS group: 100 ng/ml LPS was added to the cell culture mediums for stimulation for 24 h; (iii) AuNCs group: 50 μM of AuNCs was added to the culture medium after the treatment with LPS; (iv) BRB group: 50 μM of BRB was added after the treatment with LPS; and (v) BRB-AuNCs group: 50 μM of BRB-AuNCs was added after the treatment with LPS group.

### Cell viability

The reduction of MTT to purple formazan product analysis was used to determine the effect of BRB and BRB-AuNCs on cell survival. In the first step, the cells were seeded in a 96-well plate (4 × 10^3^). Gradient concentrations of BRB and BRB-AuNC (0, 1, 10, 50, 100 and 500 µM) were used and incubated overnight. In the second step, MTT (20 µl) was added to each well and incubate at 37°C for 4 h. Subsequently, DMSO (150 µl) was added to each well and incubated for 10 min (37°C). Finally, the absorbance was measured by a microplate reader at 490 nm.

### Confocal fluorescence imaging

RAW 264.7 cells and VSC 4.1 cells were cultured in a confocal petri dish. After washing three times with PBS, the cells were fixed with 4% PFA for 30 min. Next, the cells were washed three times with PBS and then infiltrated with 0.1% Triton X-100 for 30 min. The cells were blocked with normal goat serum for 2 h and then washed with PBS three times. Primary antibodies including anti-β-Tubulin (1:1000), anti-CD86 (1:200), anti-CD206 (1:200) and anti-Cleaved Caspase-3 (1:200) were added and incubated at 4°C overnight. On the following day, the cells were washed with PBS (3 × 5 min), and then Alexa Fluor^®^568 goat anti-mouse/rabbit IgG (1:500) and Alexa Fluor^®^488 goat anti-mouse/rabbit IgG (1:500) were added and incubated for 2 h at room temperature. The cells were rinsed with PBS (3 × 5 min) and incubated with DAPI (1:1000) for 30 min. Finally, the cells were then observed with a high-resolution confocal microscope (Leica, Heidelberger, Germany).

### Animal model and treatment

Sprague Dawley female rats (bodyweight 180–220 g, 8 weeks old) were purchased from Liaoning Changsheng Biotechnology Co., Ltd. (Benxi, China). These rats were maintained in a suitable environment, undergo a 12-h light/dark cycle at 22 ± 2°C, and provide free food and water. All experimental procedures used in this study were approved by the Animal Care and Use Committee of Jinzhou Medical University (SYXK[Liao]2019-0009). The experimental animals were divided into four groups: Sham group, Saline group, BRB group and BRB-AuNCs group. All animals were intraperitoneally anesthetized with sodium pentobarbital at 40 mg/kg, and the SCI model was established using the Allen method described earlier [22]. In short, a laminectomy was performed at the T9-T10 level and a moderate contusion was caused by dropping an impactor (diameter 2 mm, 10 g, height 25 mm) on the surface of the spinal cord. The sham group only received laminectomy. One hour after the operation, the rats were injected with BRB (10 mg/kg) or BRB-AuNCs (10 mg/kg) through the tail vein for three consecutive days. The Saline group injected the same amount of 0.9% saline.

### Western blot

Cells or spinal cord tissue 3 days after surgery were collected for western blot assay according to previous studies [2]. The following antibodies were used: anti-CD86 (1:1000), anti-CD206 (1:1000), anti-Cleaved Caspase-3 (1:1000), anti-Bax (1:1000), anti-Bcl-2 (1:1000), anti-IL-1β (1:1000), anti-IL-6 (1:1000), anti-TNF-α (1:1000), anti-β-Tubulin (1:1000), anti-GAPDH (1:1000), HRP Affinipure Goat Anti-Mouse IgG (1:10 000) and HRP Affinipure Goat Anti-Rabbit IgG (1:10 000).

### Behavior evaluation

The Basso, Beattie and Bresnahan (BBB) exercise score scale was used to evaluate the recovery of the rats’ motor function. In short, the animals were placed on the platform, and the walking and physical activities of hind limbs were observed and recorded. The scoring was divided into three parts: the first part was 0–7 points, judging the movement of the joints of the hind limbs of the animals. The second part was 8–13 points, judging the gait and coordination function of the hind limbs. The third part was 14–21 points, evaluating the fine movement of the paw during exercise. The full score was 21 points, and the experimental animals were performed on 1, 3, 7, 14, 21 and 28 days after injury.

The inclined plane test was used to assess the overall strength of the limbs. Briefly, the surface of the inclined board was matted with a 6 mm thick rubber pad, and the rats were placed in the direction perpendicular to the axis of the rats’ body and the longitudinal axis of the inclined board, and the angle between the inclined board and the horizontal plane was gradually increased until the rats can just stay on the board for 5 s. The measurement was repeated three times for each rat, and the highest score was recorded. Both trials were double-blindly scored by three well-trained experimenters.

### Histological evaluation

At 28 days after the establishment of the SCI model, the experimental animals were infused into the heart with 0.9% normal saline and 4% PFA after being anesthetized. The 0.5 cm tissue centered on the injury point was extracted and subjected to steps, such as dehydration, embedding and sectioning to make 5 μm paraffin sections. Then, staining was performed using a HE staining kit and Nissl staining solution (methylene blue). All images were obtained and observed under an optical microscope. For Nissl staining, we counted the number of ventral motor neurons (VMN) in the slices.

### Immunofluorescence analysis

Three days after the establishment of the SCI model, the experimental animals were infused into the heart with 0.9% saline and 4% PFA in sequence after being anesthetized. The 0.5 cm tissue centered on the damage point was extracted and then subjected to dehydration, embedding, slicing and other steps to make 5 μm frozen sections. After washing with PBS, the tissue was incubated in 0.3% Triton X-100 for 15 min and then blocked with 5% goat serum for 2 h. Then, the tissue was incubated with the primary antibody overnight at 4°C. Next, the tissues were incubated with the secondary antibody for 2 h at room temperature. The nuclei were stained with a DAPI solution for 15 min. The tissues were then imaged with a fluorescence microscope (Olympus, Tokyo, Japan), and the results were processed by Image J. The following antibodies were used: anti-CD11b (1:200), anti-TUBB3 (1:200), anti-Cleaved Caspase-3 (1:200), Alexa Fluor 488 goat anti-mouse IgG (1:1000) and Alexa Fluor 568 goat anti-rabbit IgG (1:1000).

### Statistical analysis

All data were expressed as mean ± standard deviation. SPSS 22.0 (Chicago, Illinois, USA) was used for statistical analysis. One-way analysis of variance or two-way analysis of variance followed by Bonferroni’s *posthoc* test was performed to compare the differences between multiple groups. The results indicated that it was performed in triplicate. For all data, *P* < 0.05 was considered statistically significant.

## Results and discussion

### Characterization of the materials

**Figure 2.**
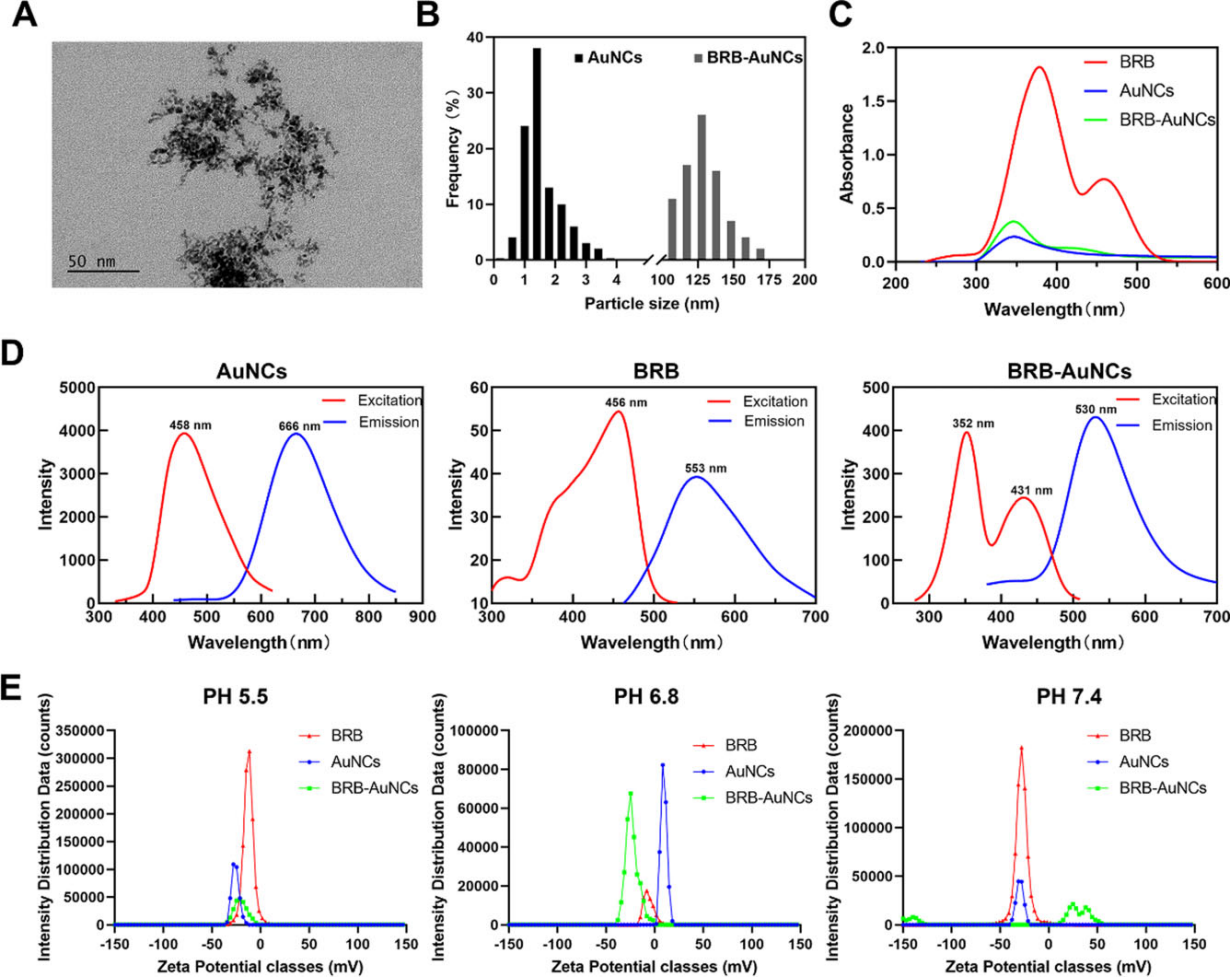
Characterization of BSA-AuNCs and BRB-AuNCs. (**A**) TEM image of BSA-AuNCs, (**B**) particle size distribution of AuNCs and hydrodynamic size distribution of BRB-AuNCs. (**C**) UV–Vis, (**D**) fluorescence excitation, emission spectra, (**E**) Zeta potential of AuNCs, BRB and BRB-AuNCs

BSA-AuNCs were characterized by TEM (Fig.  2A), which showed an average size of about 2 nm small clusters (Fig.  2B). Meanwhile, the dynamic size of BRB-AuNCs was measured as 121.982 ± 20.913 nm, indicating the conjugation of BRB aggregates and AuNCs. The optical properties of AuNCs for BRB and BRB-AuNCs were exhibited in Fig.  2C and D. Both the UV–Vis (Fig.  2C) and the fluorescence (Fig.  2D) spectra of BRB-AuNCs combined the features of AuNCs and BRB. Unlike BRB and AuNCs, which only have a single zeta potential peak, BRB-AuNCs have multiple peaks, resulting in an average potential between different peak positions. It is worth noting that only the dominant zeta potential peaks of BRB-AuNCs are switchable from negative to positive charges under different pH environments (i.e. 5.5, 6.8 and 7.4) (Fig.  2E). At pH 7.4, BRB-AuNCs have obvious peaks in the positively charged region, probably due to two reasons. First, because of the release of BRB at this Physiological environment pH, BRB-AuNCs and the released BRB aggregates exhibit variable sizes. Another reason is that BRB-AuNCs show both positive and negative charges at different surface sites. This enables BRB-AuNCs to exhibit as a zwitterionic drug [23] that will be very beneficial for the treatment of diseases. The positive charge facilitates the interaction of the drug with the negatively charged cells, while the negative charge can protect the cell from excessive damage (Fig.  2E and Table  1).

**Table 1.** Zeta potentials of AuNCs, BRB and BRB-AuNCs at different pH cultures

	AuNCs	BRB	BRB-AuNCs
PH 5.5	−25.36 ± 0.38	5.98 ± 0.63	−21.17 ± 1.56
PH 6.8	−27.63 ± 0.55	−3.57 ± 0.65	−23.87 ± 1.22
PH 7.4	−28.33 ± 0.69	−12.5 ± 0.32	−22.57±.3.60

Zeta potential (mV).

### BRB loading, release and biodistribution

**Figure 3.**
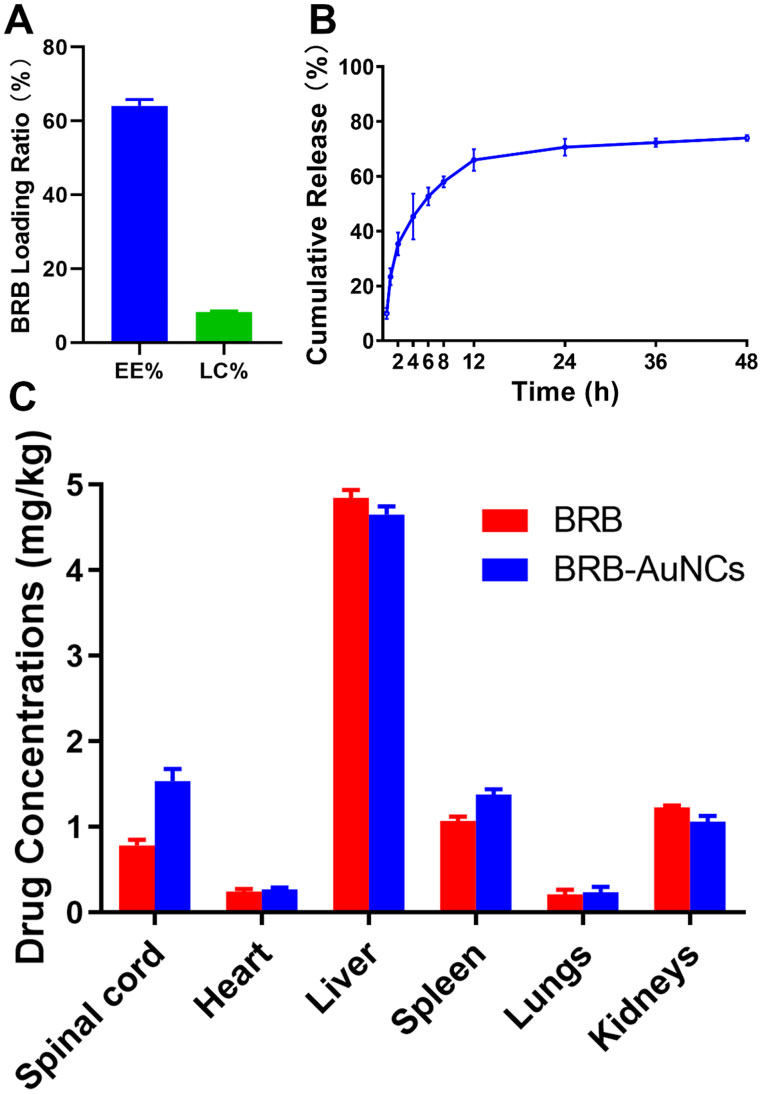
BRB Loading, release, and biodistribution. (**A**) EE% and LC% of BRB-AuNCs, (**B**) drug release of BRB-AuNCs. (**C**) BRB biodistribution *in vivo* after the administration of BRB and BRB-AuNCs, respectively

For understanding the interaction of BRB with AuNCs and the release of BRB from BRB-AuNCs, EE% and LC% were investigated according to the experiment section, which was calculated as 64.10 ± 1.69% and 8.28 ± 0.28% (Fig.  3A), respectively. BRB was found to be released from BRB-AuNCs *in vitro*. At 24 h, the cumulative release reached 70.67 ± 3.05% (Fig.  3B). To observe the distribution of BRB in the body, it was extracted from the spinal cord, heart, liver, spleen, lungs and kidneys of experimental animals after the administration of BRB-AuNCs. In the BRB-AuNCs-administered group, more BRB was distributed in the SCI area of the rats. This proves that the construction of BRB-AuNCs significantly increases the absorption rate of BRB.

### Anti-inflammatory effect *in vitro*

**Figure 4.**
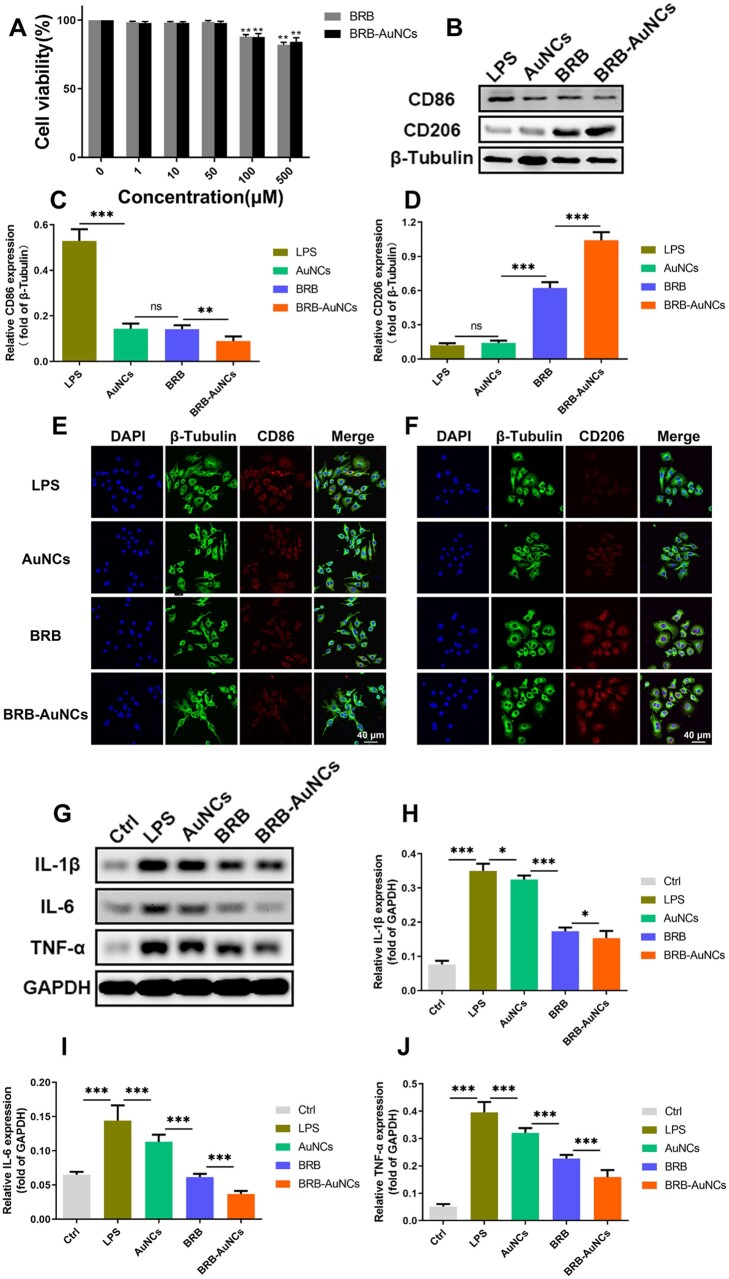
BRB-AuNCs inhibited the activation of M1 RAW264.7 cells and promoted the polarization of M2 RAW264.7 cells *in vitro*. (**A**) Effects of different concentrations of BRB and BRB-AuNCs on the proliferation of RAW264.7 cells by MTT assay. (**B–D**) Western blot examination of CD86 and CD206 protein expression and its semi-quantification analysis of RAW264.7 cells in LPS group, AuNCs group, BRB group and BRB-AuNCs group. (**E, F**) Confocal microscopy immunofluorescence analysis of CD86 and CD206 protein expression of RAW264.7 cells in LPS group, AuNCs group, BRB group and BRB-AuNCs group. Scale bars are 40 μm. (**G–J**) Western blot examination of IL-1β, IL-6 and TNFα protein expression and its semi-quantification analysis of RAW264.7 cells in the control group, LPS group, AuNCs group, BRB group and BRB-AuNCs group. Data are presented as mean ± standard deviation (*n* = 3). Statistical analysis: *P <* *0.05, **P <* *0.01, ***P <* *0.001

To determine the *in vitro* toxicity of BRB and BRB-AuNCs, the MTT method was used to detect the effects of different concentrations of BRB and BRB-AuNCs on RAW264.7 cells. As shown in Fig.  4A, when the drug concentration exceeded 100 μM, the cell activity began to decrease, but the overall survival rate was still above 80%. On the other hand, the cell viability is almost 100% when the concentration of BRB or BRB-AuNCs was lower, so 50 μM was selected for subsequent experiments. The western blot (Fig.  4B) showed the expression of CD86 in these cells increased, while the expression of CD206 decreased, which indicated that the macrophages/microglia were polarized into M1 pro-inflammatory cells after treatment with LPS *in vitro* [24]. On the other hand, the BRB group and BRB-AuNCs group enable the changing trend of the two markers more dramatically, while BRB-AuNCs most significantly increased CD86 while decreasing CD206 (Fig.  4B–D). As shown in Fig.  4E, CD86 showed strong red fluorescence under LPS stimulation; the BRB group and the AuNCs group have relatively weak red fluorescence; BRB-AuNCs group showed much weaker red fluorescence; at the same time, as shown in Fig.  4F, the expression of CD206 in the LPS group showed the weakest fluorescence; the red fluorescence of the BRB group and AuNCs group were relatively weak; BRB-AuNCs group showed bright red fluorescence. The fluorescence imaging indicated the reduced CD206 and enhanced CD86 level, and it was in good agreement with the western blot investigation, both of which represent a trend of transformation of macrophages/microglia from M1 type to M2 type.

Western blot analysis was performed to measure the levels of inflammatory factors (IL-1β, IL-6 and TNF-α). In Fig.  4G–J, the LPS group has the highest levels of three inflammatory factors. In contrast, the level of inflammatory factors in the BRB group and the AuNCs group was significantly lower. BRB-AuNCs group had the lowest level of the inflammatory factors, indicating the best anti-inflammatory ability.

The inflammatory response after SCI is considered to be the main factor that aggravates the injury, and the overexpression of effective pro-inflammatory factors is considered to be one of the main treatments for SCI [4]. M1 macrophages/microglia are macrophages that can produce pro-inflammatory cytokines [25, 26]. When the cells are stimulated, classic macrophage activation can occur, which stimulates the production of pro-inflammatory cytokines. However, the stimulating factors, such as IL-1β, IL-6 and TNF-α, were found to be downregulated, which could be beneficial to the differentiation of the M2 subgroup (Fig.  4). The downregulation of these inflammatory factors also suggested the BRB-AuNCs effectively promoted the conversion of macrophages/microglia from M1 to M2, which reduced the inflammatory response after SCI. Various nanomaterials, such as gold nanoparticles (AuNPs) were not only used as nano-carriers for anti-inflammatory drugs but also had anti-inflammatory activity themselves [27–29]. The combination of AuNPs and drugs can significantly improve efficiency because it enhances the biocompatibility of drugs and exerts a synergistic effect. AuNCs have a smaller size and are reasonable to be used as more effective carriers to improve the efficiency of BRB [20, 30].

### Anti-apoptosis effect *in vitro*

**Figure 5.**
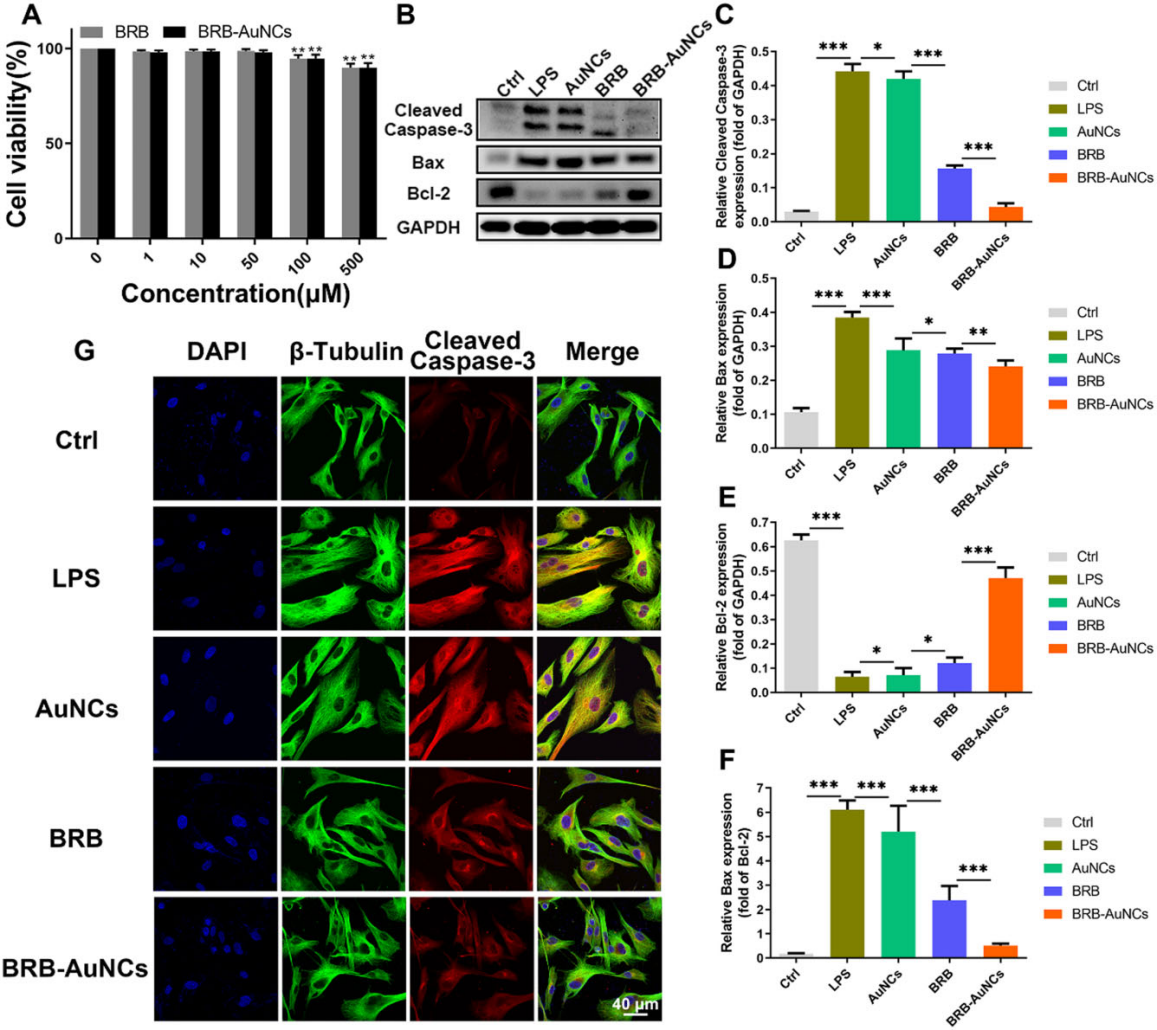
BRB-AuNCs Inhibited apoptosis of VSC4.1 cells *in vitro*. (**A**) Effects of different concentrations of BRB and BRB-AuNCs on the proliferation of VSC4.1 cells by MTT assay. (**B–F**) Western blot examination of cleaved caspase-3, bax and bcl-2 protein expression and its semi-quantification analysis of VSC4.1 cells in the control group, LPS group, AuNCs group, BRB group and BRB-AuNCs group. (**G**) Confocal microscopy immunofluorescence analysis of cleaved caspase-3 protein expression of VSC4.1 cells in the control group, LPS group, AuNCs group, BRB group and BRB-AuNCs group. Scale bars are 40 μm. Data are presented as mean ± standard deviation (*n* = 3). Statistical analysis: *P < 0.05, **P < 0.01, ***P < 0.001

To prove the anti-apoptotic effect of BRB-AuNCs *in vitro*, we selected VSC4.1 cells as an *in vitro* model. The same method was adopted to evaluate the toxicity of BRB and BRB-AuNCs on VSC4.1 cells (Fig.  5A). As shown in Fig.  5B–F, under the stimulation of LPS, the expression levels of apoptosis marker proteins Cleaved Caspase-3 and Bax increased significantly, while the anti-apoptotic protein Bcl-2 showed low levels. With the addition of BRB and AuNCs, the expression of Cleaved Caspase-3 and Bax decreased, and the expression of Bcl-2 increased, which demonstrated that BRB and AuNCs could resist apoptosis caused by LPS stimulation. However, the BRB-AuNCs group showed much more excellent anti-apoptosis ability. We also observed the expression of Cleaved Caspase-3 in each group of cells through a cell confocal microscope. As shown in Fig.  5G, the LPS group expressed strong red fluorescence. On the other hand, the red fluorescence of AuNCs group and BRB group was relatively weak, indicating that both agents inhibited apoptosis. BRB-AuNCs group showed the weakest red fluorescence, revealing that BRB-AuNCs had stronger anti-apoptotic ability than AuNCs and BRB. The fluorescence imaging of these factors was consistent with the western blot results. Overall, it can be concluded that BRB-AuNCs had an excellent anti-apoptosis effect, which was related to the strong anti-inflammatory ability of BRB-AuNCs stimulated by LPS.

Nerve cell apoptosis is the pathological change in the secondary injury stage of SCI [31]. The causes of cell apoptosis are complex, and it is often a process involving multiple injury factors and multiple apoptosis-related genes simultaneously or successively [32]. The induced pro-inflammatory cytokines [33] also lead to nerve cell apoptosis, which is the main reason for the loss of motor function and difficulty in recovery after SCI [6, 34]. BRB-AuNCs reduced the production of IL-1β, IL-6 and TNF-α, indicating their anti-inflammatory effects, thereby the apoptosis was inhibited. AuNCs play a role as a carrier to improve the biocompatibility of BRB. Additionally, our previous work found AuNCs themselves could have anti-oxidant and anti-inflammatory effects because of their (catalase) CAT-like or (Glutathione peroxidase) GPx-like activity [35]. The synergistic effect of the therapeutic effect of AuNCs themselves [36, 37] and the biocompatibility enhancement both contribute to the enhanced anti-inflammatory and anti-oxidant effect of BRB, all of which may be related to the decrease of apoptosis levels [38]. Thereafter, the recovery of damaged function after SCI is in turn expected to be recovered.

### BRB-AuNCs protects neurons and promotes motor function recovery after SCI

**Figure 6.**
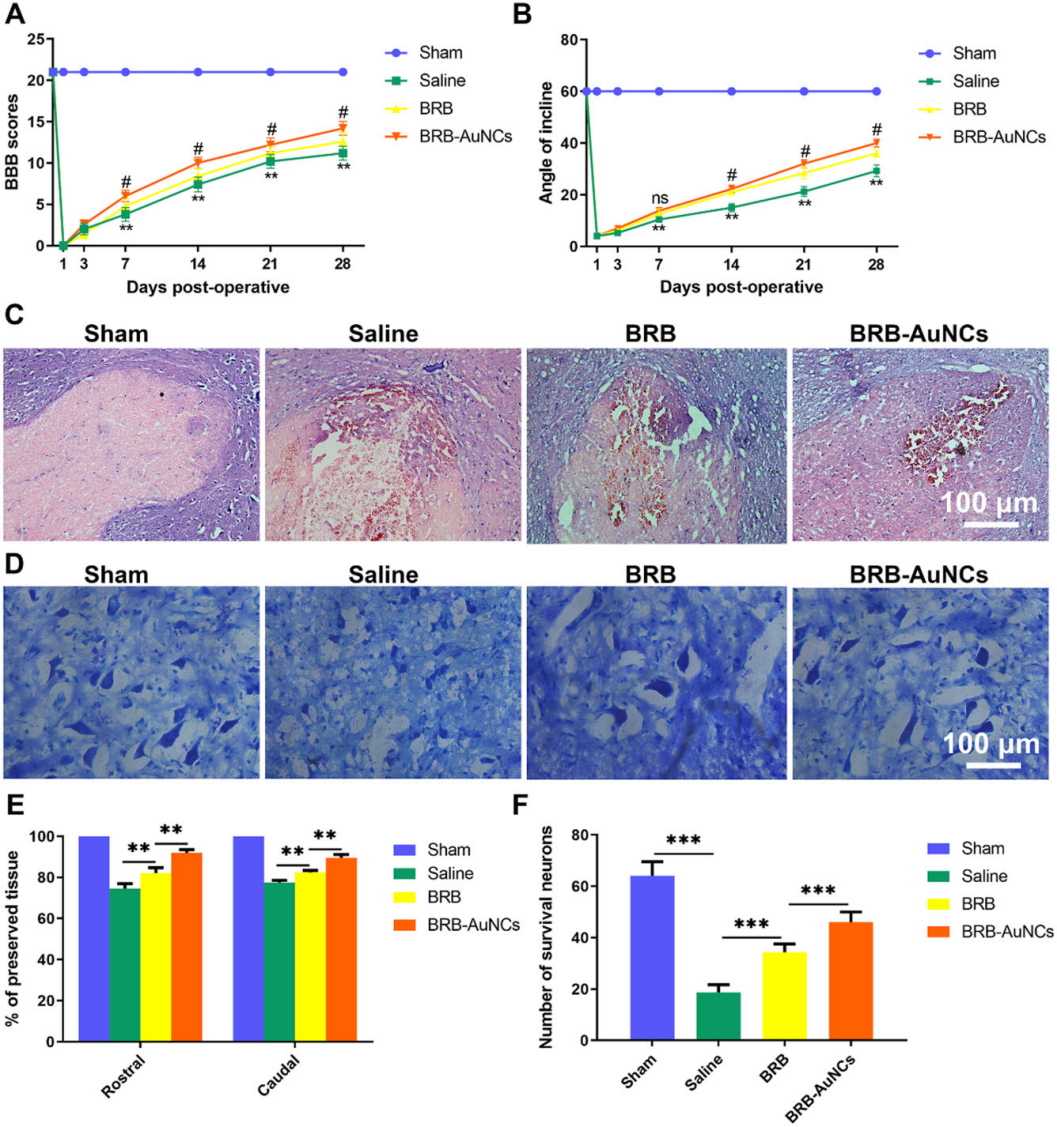
BRB-AuNCs Protect neurons and promote motor function recovery after SCI. (**A**) BBB scores of rats in each group. (**B**) Inclined plate test of rats in each group. (**C**) The HE staining of spinal cord slices in each group on the 28th day after SCI. (**D**) The Nissl staining of spinal cord slices in each group on the 28th day after SCI. (**E**) Graphic presentation of the percent of preserved tissue about the transverse area of the spinal cord on the 28th postoperative day. (**F**) Counting analysis of VMN at rostral 5 mm, caudal 5 mm and lesion site. Scale bars are 100 μm. Data are presented as mean ± standard deviation (*n* = 3). Statistical analysis: #*P* < 0.01, **P < 0.01, ***P < 0.001

We used the BBB score (Fig.  6A) and the inclined plate test (Fig.  6B) to evaluate the efficiency of the recovery of motor function in SCI rats. An insignificant difference between Days 1 and 3 (*P* ˃ 0.05) was exhibited, but starting from Day 7, with the increase of time, the BBB score of the BRB group and the BRB-AuNCs group was higher than that of the Saline group. The BRB-AuNCs group gets higher scores than the BRB. The inclined plate test shows the same trend as the BBB score. On the other hand, we used HE staining to observe changes in the damaged area. As shown in Fig.  6C, we found significant differences in histomorphology between the Sham group, the Saline group, the BRB group and the BRB-AuNCs group on the 28th day after SCI. Compared with the Sham group, the back white matter and central gray matter of the injury group were significantly damaged. Compared with the Saline group, the damage in the BRB group was reduced, and the BRB-AuNCs group decreased more significantly (Fig.  6E). On the 28th day after SCI, the number of spinal motor neurons can be seen by Nissl staining, and the average number of neurons per rat was calculated. Compared with the Saline group, the number of motor neurons in the anterior horn of the spinal cord increased significantly in the BRB and BRB-AuNCs group. And the BRB-AuNCs group has more Nissan bodies compared to the BRB group (Fig.  6D and F). These results further indicated that BRB-AuNCs reduced the death of nerve cells through its anti-inflammatory effect, thereby influencing the BBB score after SCI. And BRB modified by AuNCs has advantages over BRB alone.

The improved BBB score on each stage by the administration of BRB-AuNCs surpass many previous drugs (Table  2). This does not fully reflect the repair efficiency of SCI, but it makes us believe that BRB-AuNCs have expectant prospects for the treatment of SCI. Thus, the *in vivo* treatment effects of SCI rats were further investigated.

**Table 2.** Comparison of the motor function recovery after SCI by using different drugs

Drugs	Animal models	Motor function recovery	Other therapeutic effects	Mechanisms	Ref.
Valproic acid	Traumatic SCI rat model	Improve significantly	Inhibits the NF-kB p65 transcriptional activity and weakens the microglia-mediated central inflammatory response	Induces inflammation via STAT1 and NF-κB pathway-dependent of HDAC3	[39]
Resveratrol	Traumatic SCI rat model	Improve significantly	Enhances autophagy	Activates autophagy and inhibits apoptosis mediated by the SIRT1/AMPK signaling pathway	[40]
Melatonin	Traumatic SCI rat model	Improve significantly	Enhances autophagy	Suppress inflammation and apoptosis	[41]
Hepatocyte growth factor	Traumatic SCI rat model	Improve significantly	Enhances angiogenesis around the lesion epicenter	Promotes neuron and oligodendrocyte survival, angiogenesis, axonal regrowth, and functional recovery	[42]
Zonisamide-loaded triblock copolymer nano micelles	Spinal cord hemi-cut rat model	Improve significantly	Inhibits oxidative stress	Plays a neuron-protective role by the antioxidative effect	[43]
BRB-AuNCs	Traumatic SCI rat model	Motor function is significantly improved or even better recovery effect from the 7th day	Promotes the polarization of M1 phenotype macrophages/microglia to M2 phenotype	Triggers inflammation based on modulation of M1/M2 macrophage and reduction of apoptosis	Current work

### Anti-inflammatory effect *in vivo*

**Figure 7.**
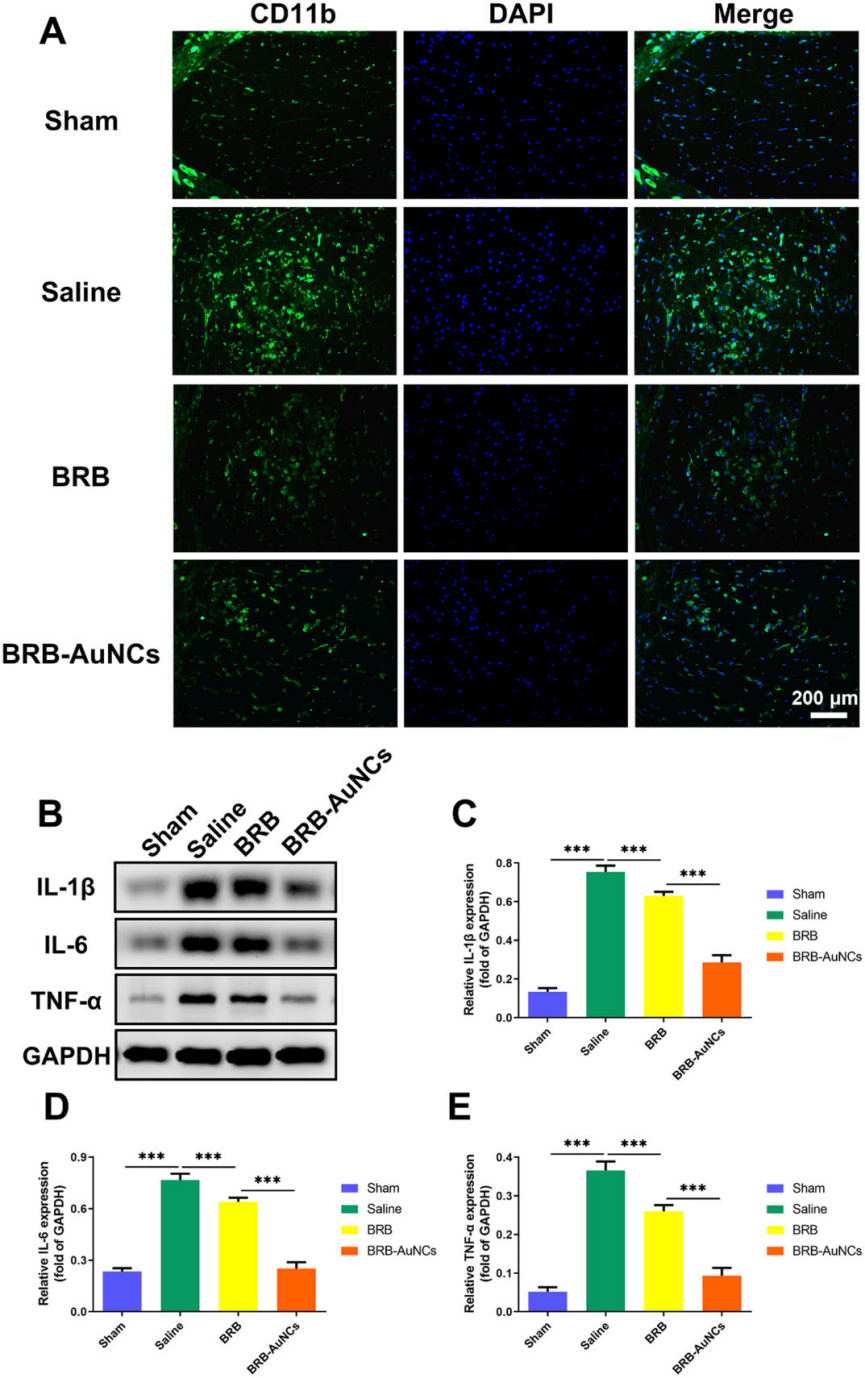
BRB-AuNCs could reduce inflammation of the injured site by inhibiting the activation of M1macrophages/microglia. (**A**) Fluorescence microscope images of spinal cord tissue slices in the sham group, saline group, BRB group and BRB-AuNCs group. (**B–E**) Western blot examination of IL-1β, IL-6 and TNFα protein expression and its semi-quantification analysis of spinal cord tissue in sham group, saline group, BRB group and BRB-AuNCs group. Scale bars are 200 μm. Data are presented as mean ± standard deviation (*n* = 3). Statistical analysis: ***P < 0.001

To study the effects of BRB-AuNCs on the polarization of macrophages/microglia after SCI, we used spinal cord tissue sections 3 days after injury to mark CD11b, a marker of macrophages/microglia. As shown in Fig.  7A, in the Saline group, we could see a large accumulation of macrophages/microglia in the damaged area, showing strong green fluorescence. This showed that inflammatory factors were released in large quantities after injury. Macrophages/microglia were activated, and the M1 phenotype was mainly expressed. At the same time, after receiving BRB-AuNCs treatment, the green fluorescence was greatly weakened, indicating that BRB-AuNCs effectively inhibited M1 phenotype macrophage/microglia polarization, and showed a better effect than simply using BRB. On the other hand, it can be seen in Fig.  7B–E that the inflammatory factors in the Sham group have almost no expression, while the Saline group has high expression. Significantly, the expression level of inflammatory proteins in the BRB-AuNCs group was greatly reduced and more obvious than that in the BRB group. These results indicated that BRB-AuNCs effectively inhibited M1 polarization and thereby reduced the level of inflammation, which was in agreement with the *in vitro* study.

### Anti-apoptosis effect *in vivo*

**Figure 8.**
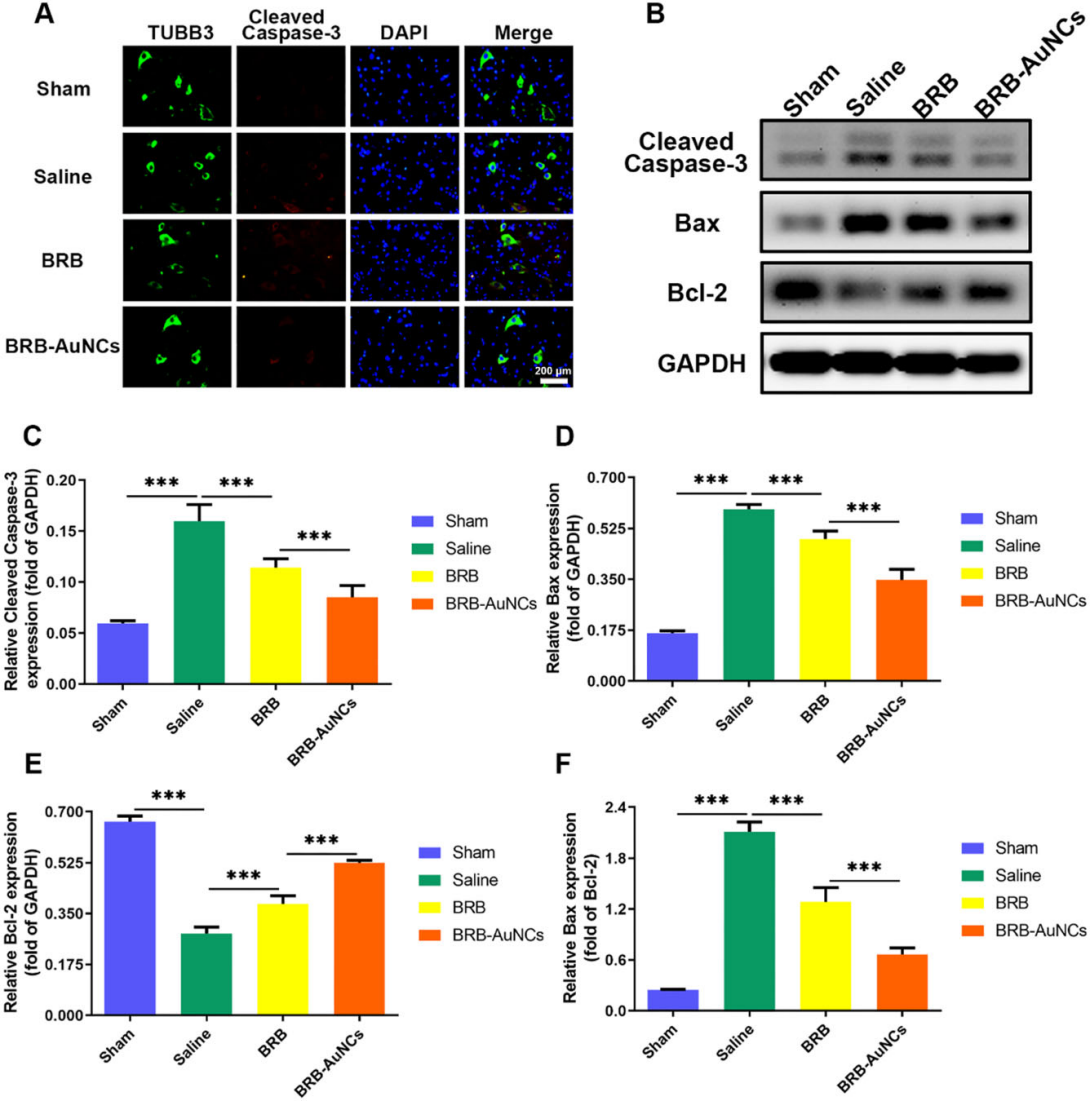
BRB-AuNCs could inhibit nerve cell apoptosis. (**A**) Fluorescence microscope images of spinal cord tissue slices in the sham group, saline group, BRB group and BRB-AuNCs group. (**B–F**) Western blot examination of Cleaved Caspase-3, bax and bcl-2 protein expression and its semi-quantification analysis of spinal cord tissue in sham group, saline group, BRB group and BRB-AuNCs group. Scale bars are 200 μm. Data are presented as mean ± standard deviation (*n* = 3). Statistical analysis: ***P < 0.001

To analyze the anti-apoptosis effect of BRB-AuNCs on nerve cells after SCI, we used fluorescent double-labeling to mark the Cleaved Caspase-3/TUBB3 in the anterior horn of the spinal cord. The motor neurons, a kind of nerve cells, are mainly located in the anterior horn of the spinal cord. It dominates the muscle activity of the limbs and is the primary center of skeletal muscle reflex. If this neuron is damaged, it will show as motor dysfunction, and it will also lead to the disappearance of some reflex activities. In Fig.  8A, it could be seen that compared to other groups, the fluorescence intensity of the Saline group increased significantly, which represented that the expression of Cleaved Caspase-3 was enhanced and the nerve cells were in a state of apoptosis. This significantly reduced the recovery of motor function in patients with SCI. The red fluorescence intensity of the BRB-AuNCs group was the lowest compared with the Saline group and the BRB group, which indicated that BRB-AuNCs have an anti-apoptosis effect. In addition, the results of the western blot (Fig.  8B) analysis further confirmed the above results. Figure  8B–F shows the expression level of apoptosis-related proteins (Cleaved Caspase-3, Bax and Bax/Bcl-2) decreased significantly with the treatment of BRB-AuNCs. The *in vivo* results were in good agreement with the *in vitro* investigations, which further illustrates the anti-apoptotic ability of BRB-AuNCs.

### Toxicology *in vivo*

**Figure 9.**
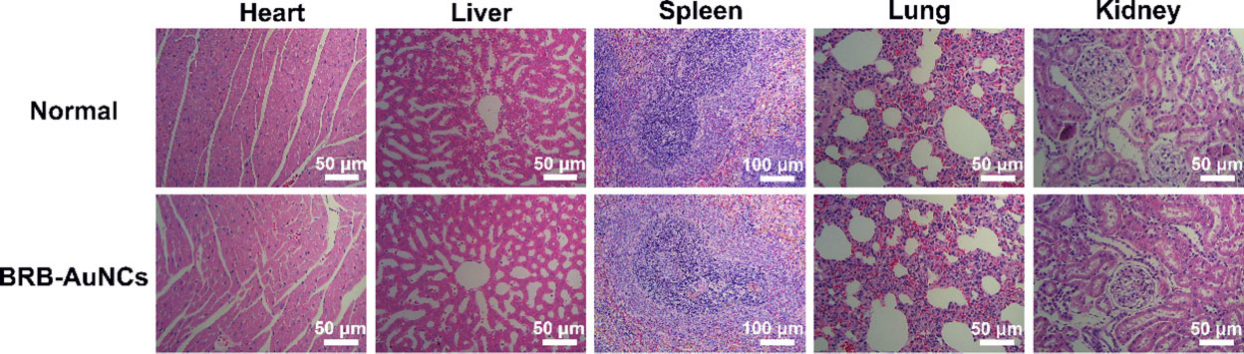
BRB-AuNCs had insignificant toxicity *in vivo*. HE staining provided photos of the heart, liver, spleen, lung and kidney. Scale bars are 50 and 100 μm, *n* = 3

To understand the safety of BRB-AuNCs *in vivo*, the HE staining analysis was performed on various organs and tissues (heart, liver, spleen, lung and kidney) of rats injected with BRB-AuNCs (Fig. 9). It can be seen that there was no organ damage after the use of BRB-AuNCs compared with the normal control group. This shows that BRB-AuNCs are promising to be developed as safe drugs with insignificant toxicity.

## Conclusions

This work employs AuNCs to activate the potentially powerful therapeutic effects of BRB. BRB-AuNCs were facilely developed, which was found to significantly reduce inflammation based on the polarization of M1 phenotype macrophages/microglia to M2 phenotype as well as alleviating neuronal apoptosis. Most excitingly, compared with many other drugs, the motor function and inflammation of SCI rats have been improved. This work opens an avenue for the therapy of SCI by BRB-AuNCs. It also provides a new strategy by using AuNCs to improve the drug utilization of BRB, which is also promising to treat inflammation caused by many diseases.

## Funding 

This work was supported by the National Natural Science Foundation of China (NSFC) (NO. 81871556 and 82072165) and Liaoning Revitalization Talents Program (NO. XLYC1902108).

*Conflict of interest statement*. None declared. 
